# Massive Fluid Requirements and an Unusual BUN/Creatinine Ratio for Pre-Renal Failure in Patients with Cholera

**DOI:** 10.1371/journal.pone.0007552

**Published:** 2009-10-26

**Authors:** Muhammad Tariq, Murtaza Memon, Asif Jafferani, Sana Shoukat, Saqib Ali Gowani, Rabeeya Nusrat, Mehmood Riaz, Junaid Patel, Bushra Jamil, Raymond A. Smego

**Affiliations:** 1 Department of Medicine, Aga Khan University, Karachi, Pakistan; 2 MBBS, Aga Khan University, Karachi, Pakistan; HelmholtzZentrum München, Germany

## Abstract

**Background:**

Cholera is an important infectious cause of secretory diarrhea. The primary symptom of infection is the sudden onset of watery diarrhea with subsequent volume depletion causing renal insufficiency. The objective of this research is to study the level of dehydration at presentation and subsequent fluid management in patients with cholera.

**Methods:**

This study was conducted on 191 patients of Cholera admitted at a tertiary care hospital in Karachi, Pakistan during the period of 5 years. Medical charts were evaluated retrospectively for initial hydration status, baseline lab investigations on admission and discharge and fluid therapy given to all the patients while their stay in the hospital and the data was analyzed on SPSS 15.0.

**Results:**

Out of the 191 patients, 83(43%) were males and 108 (57%) were females with mean age of 42.3 years (SD±18.34). The average duration of symptoms was 3.75 days (SD±2.04). Of 191 patients, 175 (92.1%) presented with dehydration, 80 (42.3%) were given Ringer's Lactate (R/L) + Normal Saline (N/S), 45 (24%) patients were given R/L + N/S + Oral Rehydration Therapy (ORS), 27 (14.3%) of the patients were kept on R/L only and remaining were given various combinations of R/L, N/S, ORS and Dextrose Saline (D/S). On admission mean Blood Urea Nitrogen (BUN) was 24.54 (SD±16.6), mean creatinine was 2.47 (SD±2.35) and mean BUN/Creatinine ratio was 11.63 (SD±5.7).

**Conclusion:**

Aggressive fluid rehydration remains the cornerstone of management of cholera. Instead of presenting with a classical BUN/Creatinine ratio of >20∶1, patients with pre-renal failure in cholera may present with a BUN/Creatinine ratio of <15∶1.

## Introduction

Cholera has long been known as a cause of diarrhea [1]. This acute diarrheal illness is caused by certain serotypes of Vibrio Cholerae [2]. *V. cholerae* is traditionally classified by O group (with >150 O types currently recognized in the widely used Sakazaki grouping system) and by biotype (classical and El Tor) and serotype (Ogawa, Inaba, and, rarely, Hikojima)[3]. Contaminated food and water is the mode of transmission for this infection [4]. It causes massive fluid loss by toxin mediated hypersecretion of water and chloride ion [5]. The incubation period of cholera can range from several hours to 5 days [6]. The common symptom associated with cholera is acute, painless, voluminous, watery diarrhea and effortless vomiting, the diarrhea is sometimes referred as “rice water stools”[7], [8]. It is diagnosed by Stool culture and agglutination of vibrios with specific sera [9]. The treatment management of cholera revolves around the oral or Intravenous (I/V) replenishment of the lost fluid, which is the mainstay therapy for these patients [10], [11]. Antimicrobial therapy shortens the course of the illness. Several active antibiotics against V. Cholera have been used including tetracycline, ampicillin, ciprofloxacin and trimethoprim- sulfamethoxazole [12]. Untreated cholera cases can have fatality rates above 50% [13]. The objective of our study is to determine the BUN/Creatinine ratio in cholera patients with dehydration and renal insufficiency and amount of fluid replacement therapy given in a tertiary care hospital in Pakistan.

## Materials and Methods

### Ethics Statement

This study was conducted according to the principles expressed in the Declaration of Helsinki. The study was approved by the Ethics Review Committee of the Department of Medicine of the Aga Kahn University.

### Methods

Medical records of 191 patients diagnosed with Cholera in the last 5 years at Aga Khan University Hospital, Karachi, were evaluated retrospectively. Patients included in the study were identified using proper diagnostic criteria which included patients presenting with classical signs and symptoms of cholera along with positive lab cultures.

Medical charts were evaluated for baseline investigation including initial hydration status, BUN on admission, creatinine on admission, BUN/creatinine ratio on admission, severity of the illness on presentation, and in- hospital investigation which included 24 hourly fluid administration, types of fluid administrated, hydration status on discharge, BUN on discharge, creatinine on discharge, BUN/creatinine ratio on discharge, duration of hospital stay, causative agents (serotype), stool cultures and clinical outcome of the patients.

The data was analyzed using SPSS 15.0 using basic frequencies and chi-squares to define the p-values.

## Results

Out of the 191 patients 83 (43%) were males and 108 (57%) were females with mean age of 42.3±18.34 years. The average duration of symptoms was 3.75 (SD±2.04) days. From all the patients, 175 (92.1%) presented with dehydration and of those, 7.5% (14), 62.0% (116), 30.5%(57) fell in the clinical category of mild, moderate and severe dehydration respectively. Mean hospital stay of the patients was 3.83 days (SD±2.785). Among species, majority were found to belong to the Ogawa species 75.7% (137) followed by 0139 species 24.3% (44). Rectal tube was used in 94.1% (174) of the patients during the hospital stay to monitor fluid loss. A total of 189 patients were treated with antibiotics during the course. 31.4% (60) patients developed acute renal failure as a complication which resolved at an average of 2.1 days (SD±3.3).

Regarding fluid resuscitation, 80 (42.3%) were given R/L + N/S, 45 (24%) patients were given R/L + N/S + ORS, 27 (14.3%) of the patients were kept on R/L only and remaining were given various combinations of R/L, N/S, ORS and D/S. (See Table 1). An average of 24826 mL (SD±15998) was given to the patients under fluid therapy during an average stay of 3.8 days (SD±2.8). 9998.5 mL (SD±5252) was the average fluid administered during first day of hospital stay. (See Table 2).

**Table 1:** Type of fluid therapy adminstered.

	Frequency	Percent
N/S	7	3.7
R/L	27	14.1
R/L+ORS	19	9.9
R/L+N/S	80	41.9
N/S+ORS	5	2.6
N/S,D/S,R/L	3	1.6
N/S+R/L+ORS	45	23.6
D/S+R/L	3	1.6
ORS	2	1.0
**Total**	**191**	**100.0**

N/S  =  Normal saline.

R/L  =  Ringer's lactate.

ORS  =  Oral Rehydration Solution.

D/S  =  Dextrose Saline.

**Table 2:** Quantity of fluids given.

	Minimum	Maximum	Mean	Std. Deviation
Fluids given in 1st 24 hrs	1800.0	38000.0	9998.5	5252.1
Total fluids administered	1800.0	40250.0	24826.8	15998.0

On admission, mean BUN was 24.54 (SD±16.6), mean creatinine was 2.47 (SD±2.35) and mean BUN/Creatinine ratio on was 11.63 (SD±5.7). On Discharge average BUN was 11.97 (SD±10.81) and average Creatinine on discharge was 1.18 (SD±1.28). At presentation, the BUN/Creatinine ratio of patients who developed Acute Renal Failure (ARF) was significantly lower than the BUN/Creatinine ratio of the patients without ARF (p value 0.04). However, the ratio was not significantly different at discharge (p value 0.81). No patient expired during the hospital stay. (See Table 3)

**Table 3:** Blood Urea Nitrogen and Creatinine levels and ratios.

	Minimum	Maximum	Mean	Std. Deviation
**BUN**				
On Admission	3.0	132.0	24.5	16.6
At Discharge	1.0	78.0	12.0	10.8
**Creatinine**				
On Admission	0.5	25.0	2.5	2.4
At Discharge	0.5	10.0	1.2	1.3
**BUN Creatinine Ratio on Admission**	1.0	31.4	11.6	5.7
**BUN Creatinine Ratio on Discharge**	0.57	42	11.72	7.9

## Discussion

Cholera still remains a cause of severe diarrhea in third world countries with poor sanitation conditions. It remained one of the common cause of mortality until late 1960s when the role of ORT (oral rehydration therapy) was established after the discovery of intact sodium-glucose intestinal co-transporter [12]. The disease is characterized by a devastating watery diarrhea which leads to rapid dehydration, and death occurs in 50 to 70% of untreated patients [14]. Despite availability of Oral Rehydration therapy for more than 3 decades the severity of diarrhea in Cholera can be estimated from 2.5 million deaths claimed per year globally [15]. The main cause of death in patients with cholera is hypovolemic shock caused by severe dehydration.

As a result of intravascular fluid depletion and decreased effective circulating volume, the blood flow to kidneys is impaired and this leads to Pre-renal failure. Pre-renal failure can be distinguished from Intra- renal failure by BUN to creatinine ratio, Fractional excretion of Sodium and Fractional Excretion of Urea nitrogen [16]. In Pre- renal failure the BUN to creatinine ratio rises from a normal level of 10∶1 to more than 20∶1. As we see in our study the average fluid deficit replaced in 3.83 days was around 25 liters but even with such deficit we noticed that the patients had an average BUN to creatinine ratio of 11.63 at the time of hospital admission. The outcome of Pre-renal failure is its rapid response to fluid therapy, and in our study the average serum creatinine on admission was 2.47 which came down to 1.18 at the time of discharge.

The disproportionate rise in BUN in pre-renal failure (due to volume depletion) is that it is due to increased tubular urea reabsorption, so the urea clearance falls more than the creatinine clearance. There is also an effect of anti-diuretic hormone, which promotes urea reabsorption.

The 20∶1 rule for BUN/Creatinine ratio in pre renal failure may only be an approximation – there may be lots of exceptions. It is more reliable if the patient is in a fairly steady state, rather than having a very acute problem. The increase in BUN with dehydration takes a while – perhaps these patients are so acutely dehydrated that they have not had enough time for the urea to reach equilibrium. We think this is the most important reason that the BUN/creatinine ratio is not higher. Also availability of Fractional Excretion of Sodium (FeNa) would have made the analysis more clear.

The baseline BUN/Creatinine ratio must also be important, and it would be lower than usual if these patients are consuming low protein intakes. We realize that the ratio was 10 at discharge, but it might have fallen further in a few more days.

Finally, it may be possible if urea is lost from the gut more rapidly than creatinine in cholera.

Further larger prospective studies are needed to study urea metabolism in pre-renal failure in cholera and the effects of cholera toxin on intestinal urea transport.

Limitations of the study included a small sample size from a tertiary care hospital, which was not at all representative of the general population. As it was a retrospective study, due to unavailability of all records, there was an inability to analyze electrolytes and there relation with the BUN/Creatinine ratio for the development of pre renal failure. Further well designed prospective studies of larger sample size need to address these limitations.

From this study we conclude that the role of rapid and aggressive fluid therapy remains crucial not only as a life saving measure in the management of Cholera but rapid fluid therapy may reverse the impending acute renal failure caused by severe dehydration. Moreover, the classical BUN/Creatinine ratio for pre renal failure of >20∶1 is missing in our patients, and they may present with a BUN/Creatinine ratio of <15∶1.
